# Physiological and electrophysiological characteristics of continuously cropped *Pinellia ternata* and a novel electrical information-based monitoring approach for its cropping obstacles

**DOI:** 10.3389/fpls.2025.1614478

**Published:** 2025-08-04

**Authors:** Cheng Zhang, Jing Liu, Zelan Wang, Xiaona Lu, Liangyu Dai, Chenglin Tang, Ye Hang, Bing Tian

**Affiliations:** ^1^ School of Public Health/Key Laboratory of Endemic and Ethnic Diseases, Ministry of Education and Key Laboratory of Medical Molecular Biology of Guizhou Province, Guizhou Medical University, Guiyang, Guizhou, China; ^2^ Guizhou Agricultural Ecology and Resource Protection Station, Agriculture and Rural Affairs Department of Guizhou Province, Guiyang, Guizhou, China; ^3^ Guizhou Crop Technology Extension Station, Agriculture and Rural Affairs Department of Guizhou Province, Guiyang, China

**Keywords:** *Pinellia ternata*, electrophysiological characteristics, continuous cropping obstacle, monitoring approach, electrical information

## Abstract

Continuous cropping obstacles drastically inhibit plant growth, medicinal composition, and tuber productivity of *Pinellia ternata*, thereby restricting its industrial development. There are few systematic reports on the physiological and electrophysiological responses of *P. ternata* under continuous cropping beyond 2 years, and effective obstacle monitoring methods remain undeveloped. Based on the results, continuous cropping for 1~4 years clearly inhibited morphological development, emergence rate, photosynthesis, antioxidant enzyme activities, and medicinal composition of *P. ternata*, while increasing its sprout tumble rate and resistance-related substance contents, as well as reducing tuber yield by 23.84%~59.20%. Meanwhile, continuous cropping could dramatically inhibit its intracellular water metabolism, nutrient transport, metabolic activity, and intrinsic capacitance (IC), while increasing its intrinsic resistance (IR), impedance (IZ), inductive reactance (IXL), and capacitive reactance (IXc). Additionally, these inhibiting or promoting effects intensified with the increasing duration of continuous cropping. Reliably, the comprehensive growth index (CGI) and continuous cropping obstacle index (CCOI), based on the plant’s electrical information, exhibited good correlations with the physiological and electrophysiological parameters, which accurately and rapidly characterized the growth and continuous cropping obstacle status of *P. ternata*. This study systematically described the physiological and electrophysiological characteristics of *P. ternata* under perennial continuous cropping, developed a novel monitoring approach based on the plant’s electrical information, and provided new insights for monitoring and mitigating its continuous cropping obstacle.

## 1 Introduction

*Pinellia ternata* (Thunb.) Breit., a traditional Chinese herb of the *Araceae* family, is widely cultivated commercially in China (Juneidi et al., 2020; Li et al., 2021). Its tuber is rich in many medicinal compounds, including alkaloids, polysaccharides, nucleosides, organic acids, flavonoids, and phenolics, and has high economic and medicinal value (Duan et al., 2019; Xue et al., 2021). Many pharmacological and medical experiments indicated that it is widely applied as an anti-insomnia, antitussive, antitumor, antiemetic, anxiolytic, analgesic, hematic fat-reducing, and liver-protecting agent (Guo et al., 2022; Lin et al., 2019; Xu et al., 2018). Recently, as a revitalizing rural agricultural industry, it has been broadly cultivated in Gansu and Guizhou provinces in China, with cultivation areas of over 1,333 and 4,000 hm^2^, respectively (Li et al., 2021). Nevertheless, the growth, development, quality, and yield of *P. ternata* were continuously inhibited, and its diseases and pests gradually intensified after long-term continuous cropping in the same soil (Chen et al., 2023; He et al., 2019a, b; Zhao et al., 2021; Zhang et al., 2021). Moreover, this soil was gradually acidified, and its nutrient or microbial community structure was continuously altered or unbalanced (Deng et al., 2025; He et al., 2019a, b; Zhao et al., 2021). Accordingly, continuous cropping obstacle drastically inhibits the morphological development, phytochemical quality, and tuber productivity of *P. ternata* and restricts its industry development. Researchers have carried out many studies on the physiological and biochemical responses, occurrence mechanisms, and mitigation measures of continuous cropping obstacle in *P. ternata* (Chen et al., 2023; Wang et al., 2025; He et al., 2022; Zhang et al., 2023).

In these reports, various mitigation measures have been recommended. For example, Hang et al. (2018) indicated that *Glycine max* intercropping improved the tuber yield of continuous cropping *P. ternata*, and *Capsicum annuum* L. intercropping enhanced its quality. He et al. (2019a) indicated that rotation could remediate its soil microbial community. Chen et al. (2023) found that chitosan spraying enhanced the plant growth, tuber yield, and phytochemical composition of continuous cropping *P. ternata*, and the associated application of plant growth regulators and a seed dressing agent could alleviate its continuous cropping stress (Tian et al., 2024). For its occurrence mechanisms, Tang et al. (2018) found that butylated hydroxytoluene was the main allelochemical component (60.35%) of *P. ternata*. Xiao and Luo (2022) demonstrated that 0.10~400 mg L^−1^ butylated hydroxytoluene exposures induced differential gene expression in its tubers. Hang et al. (2023) then reported that its physiological and transcriptomic responses were affected under continuous cropping. Subsequently, Zhang et al. (2023) found that *Bacillus cereus* WL08 immobilized in charcoal could degrade butylated hydroxytoluene in the planting soils of *P. ternata* and alleviate its continuous cropping obstacle. Recently, Wang et al. (2025) reported that a microbial agent composed of microbes with the functions of allelochemical degradation, phosphate and potassium solubilization, and nitrogen fixation could improve soil quality, as well as microbial community structure and function in continuous cropping of *P. ternata*. However, there are few systematic reports on the physiological, biochemical, and electrophysiological responses of *P. ternata* under continuous cropping beyond 2 years. For instance, Hang et al. (2023) only reported that continuous cropping for 2 years could significantly reduce the chlorophyll content and antioxidant enzyme activities of *P. ternata* and increase its reactive oxygen species and malondialdehyde. Meanwhile, the assessment of continuous cropping obstacle severity in *P. ternata* relies primarily on lagging approaches such as visual observation and postharvest quality and yield testing. However, the predictive, real-time, and rapid monitoring methods remain undeveloped and unreported in current research.

The electrophysiological activities occur throughout almost all life processes in plants, such as charge separation, electron movement, and the transport of dielectric substances (Hedrich et al., 2016; Nguyen et al., 2018; Szechyńska-Hebda et al., 2017). A plant’s electrical information is regarded as the fastest physiological response to abiotic or biotic stimuli, such as salt, drought, cold, diseases, pests, etc (Favre et al., 2011; Gallé et al., 2015; Sukhov et al., 2015). In recent reports, as a momentous finding in plant physiology, the theoretical relations between leaf resistance (*R*), impedance (*Z*), inductive reactance (XL) or capacitive reactance (Xc), and clamping force (*F*) were uncovered as *R*, *Z*, XL or Xc = *y* + *k_e_* ^(-bF), according to bioenergetics, for the first time (Zhang et al., 2020, 2021a, 2021b). Dexterously, when *F* = 0 (i.e., natural state), and *e*^0 = 1, then the intrinsic *R*, *Z*, Xc, and XL can be undamaged, real-time, rapidly, and accurately monitored as: IR, IZ, IXL, or IXc = *y* + *k*. Intrinsic capacitance (IC) can then be calculated from IXc. Many studies report that these electrical parameters were commonly used to evaluate various physiological statuses (Xing et al., 2021, 2022; Zhang et al., 2015). For example, Zhang et al. (2021a, 2021b) indicated that *R*, *Z*, XL, and Xc could be applied to evaluate the nutrient transport and plunder capacities in plants, and Xing et al. (2022) demonstrated that *Z* could manifest the intracellular water transport rate in plant leaves. Recently, Tian et al. (2024) found that co-application of epihomobrassinolide and thiamethoxam·flutolanil·azoxystrobin could effectively decrease the IR, IZ, IXc, and IXL of *P. ternata* and enhance its IC, intracellular water metabolism, nutrient transport, and metabolic activity. In this way, it is worth studying whether the plant’s electrophysiological information can be used to predict the occurrence of continuous cropping obstacles in *P. ternata*.

In this study, the physiological responses of *P. ternata* under continuous cropping for 0~4 years were first evaluated. Simultaneously, its electrophysiological responses were investigated. Subsequently, the comprehensive growth index (CGI) and continuous cropping obstacle index (CCOI), based on electrical information, were defined and applied to characterize the occurrence degree of continuous cropping obstacles in *P. ternata*. This work aims to systematically reveal the physiological and electrophysiological characteristics of continuously cropped *P. ternata* and to develop a novel approach for rapidly monitoring the occurrence of continuous cropping obstacles.

## 2 Materials and methods

### 2.1 Tuber seed and experimental field

The variety name of *P. ternata* tuber seeds was “Hemayu #1”, and their diameters ranged from 0.8 to 1.2 cm. This batch of tuber seeds was purchased from Hezhang Mountain Efficient Agricultural Technology Co. Ltd. (Bijie, Guizhou, China) and was harvested in 2023. At harvest, tubers with diameters ≥ 1.2 cm were classified as medicinal materials, while those with diameters < 1.2 cm were classified as seeds. The field experiment base of *P. ternata* was located in Huaxi District, Guiyang City, Guizhou Province, China, and the experimental plots contained soil with 0~4 years of continuous *P. ternata* cultivation history. The experimental field was specifically used during the fifth continuous cropping season.

### 2.2 Different continuous cropping year experiment

The experiment comprised five treatments based on the duration of continuous cropping: 0 years (CC0), 1 year (CC1), 2 years (CC2), 3 years (CC3), and 4 years (CC4). All treatments were implemented in the same experimental field through progressive annual advancement. Based on pre-established cultivation by the research group, a cumulative 4-year continuous cropping system was developed, and the present research was conducted during the fifth cultivation year. Notably, no intervening crops were planted between successive years of continuous cropping. Each treatment was replicated three times in randomized plots. Raised beds were constructed with dimensions of 1.0 m in length × 4.0 m in width and 0.2 m interbed spacing, yielding a plot area of 4 m^2^. P*. ternata* seeds were broadcast at a density of 150 kg per 667 m^2^ on 20 March, followed by coverage with 5.0 cm of soil. The seed specifications were roughly the same, with diameters ranging from 0.8 to 1.2 cm. Harvest was performed on 15 September after natural plant senescence.

### 2.3 Agronomic trait determination

Ten *P. ternata* plants from the south, north, east, west, and center positions of each plot were randomly chosen for measuring leaf width and length, stem diameter, and plant height using a vernier caliper or ruler at the full seedling stage (6 May), vigorous growth stage (21 May), and sprout tumble stage (15), following the methods of Tian et al. (2024). Leaf area was calculated using the leaf area coefficient (0.666) method (Chen et al., 2023) (Equation 1).


(1) Leaf area=0.666×leaf length×leaf width

#### 2.3.1 Emergence rate

The number of plants in the 1-m^2^ raised bed at the center of each plot was recorded weekly after *P. ternata* began to emerge, following the methods of Tian et al. (2024).

#### 2.3.2 Sprout tumble rate

The number of inverted seedlings in the 1-m^2^ raised bed at the center of each plot was recorded during the sprout tumble stage, following the methods of Tian et al. (2024) as Equations 2, 3.


(2) $$\begin{array}{c} \text{Emergence rate}\\ =\text{maximum number of seedlings}/\text{theoretical number of seedlings} \end{array}$$

(3) $$\begin{array}{c} \text{Sprout tumble rate}\\ =\text{inverted seedling number}/\text{total seedling number} \end{array}$$

### 2.4 Photosynthesis measurement

Ten *P. ternata* plants in the same positions as **Section 2.3** were selected on the vigorous growth stage (21 May) for determining their chlorophyll and photosynthetic capacity. The chlorophyll SPAD value of fully expanded leaves was measured by a SPAD-502 Plus meter. Moreover, their photosynthetic and transpiration rate, stomatal conductance, intercellular carbon dioxide concentration, and water use efficiency were measured using a portable LI-6400XT photosynthesis determination system (LI-COR Inc., Lincoln, NE, USA) at 8:00~10:00 a.m., and the photosynthetically active radiation was 1,000 µmol m^−2^ s^−1^.

### 2.5 Leaf resistance determination

*P. ternata* plants from the same positions as described in **Section 2.3** of each plot were collected during the vigorous growth stage (21 May), and fresh, fully expanded leaves were used to determine their assistance of *P. ternata* plants were used to determine their resistance abilities (Zhang et al., 2022, 2024). Soluble sugar, malondialdehyde, and proline contents were measured by the anthrone colorimetric, thiobarbituric acid, and ninhydrin colorimetry methods, respectively. Additionally, superoxide dismutase, catalase, and peroxidase activities were measured using the nitrogen blue tetrazole, potassium permanganate titration, and guaiacol approaches, respectively. Briefly, the specimens were processed using appropriate reagents, and the resultant supernatant obtained after centrifugation was analyzed. Enzyme activity was expressed as U g^−1^ min^−1^ FW.

### 2.6 Electrophysiological information determination

During the vigorous growth stage (21 May), fully expanded leaves from *P. ternata* plants occupying equivalent nodal positions across experimental plots were sampled to assess their bioelectrical dynamics, intracellular water metabolism, nutrient transport, and metabolic activity parameters (Tian et al., 2024, 2025). Previous reports demonstrated that the theoretical relationships between leaf resistance (*R*), impedance (*Z*), inductive reactance (XL), capacitive reactance (Xc), and clamping force (*F*) were proved as *R*, *Z*, XL or Xc = *y* + *k_e_* ^(−bF) according to Nernst equation (Zhang et al., 2020, 2021a, 2021b). The fresh, fully expanded leaves of each plant were sampled and soaked in water for 30 min. Next, *R*, *Z*, XL, and Xc under different clamping forces (1.139 N, 2.149 N, 3.178 N, 4.212 N, and 5.245 N) were measured, and the corresponding parameters were obtained from their fitting equations, respectively. When *F* = 0 (i.e., natural state), *e*^0 = 1, and then the intrinsic *R*, *Z*, XL, and Xc were calculated as Equation 4:


(4) IR, IZ, IXL or IXc=y+k

IC could then be calculated as Equation 5:


(5) $$\text{IC}=\frac{1}{2\text{πfIXc}}$$

Where *π* = 3.1416, and *f* is the frequency.

Additionally, the intracellular water-holding capacity (IWHC), intracellular water-holding time (IWHT), water or nutrient transfer rate (WTR or NTR), nutrient flux per unit area (UNF), nutrient transport capacity (NTC), active transport flow of nutrients (NAF), nutrient active transport capacity (NAC), metabolic flow (MF), metabolic rate (MR), and metabolic activity (MA) were monitored as described by Tian et al. (2024, 2025) as Equations 6–17:


(6) $$\text{IWHC}=\sqrt{{\left(\text{IC}\right)}^{3}}$$

(7) IWHT=IC×IZ

(8) $$\text{WTR or NTR}=\frac{\text{IWHC}}{\text{IWHT}}$$

(9) $$\text{UNF}=\frac{\text{IR}}{\text{IXc}}+\frac{\text{IR}}{\text{IXL}}$$

(10) NTC=UNF×NTR

(11) $$\text{UAF}=\frac{\text{IXc}}{\text{IXL}}$$

(12) NAC=UAF×NTR

(13) $$\text{MF}=\frac{1}{\text{IR}\times \text{IZ}\times \text{IXc}\times \text{IXL}}$$

(14) MR=NTR×NAC

(15) $$\text{MA}=\sqrt[{6}]{\text{MF}\times \text{MR}}\;$$

Furthermore, normalized IWHC, IWHT, NTC, NAC, and MA were used to characterize the CGI of *P. ternata.*


(16) $$\text{CGI}=\sqrt[{5}]{\text{IWHC}\times \text{IWHT}\times \text{NTC}\times \text{NAC}\times \text{MA}}$$

Meanwhile, the CGI of healthy *P. ternata* plants (i.e., with 0 years of continuous cropping) was used as the reference baseline, and the severity of continuous cropping obstacle was classified into 10 scales, using CGI to characterize its CCOI.


(17) $$\text{CCOI}=10(1-\frac{{\text{CGI}}_{\text{i}}}{{\text{CGI}}_{\text{R}}})$$

CGI*_i_* was the comprehensive growth index of *P. ternata* with continuous cropping for 1~4 years, and CGI*_R_* was the comprehensive growth index of *P. ternata* with no continuous cropping.

### 2.7 Quality and yield determination

The underground tuber organs of *P. ternata* were systematically excavated from all experimental plots on 15 September, and their medicinal composition and tuber productivity were evaluated following the methods of Tian et al. (2024). Tubers meeting the critical morphometric threshold (diameter ≥ 1.2 cm) were classified as commercial-grade materials, while those below this threshold (< 1.2 cm) were designated as seed tubers. Their weight was then measured using a balance. Quantification of water, ash, extractum, and butanedioic acid in *P. ternata* tubers was conducted in strict accordance with the standardized analytical protocols stipulated in the Chinese Pharmacopoeia Commission (2019).

### 2.8 Statistical analysis

Statistical significance was determined using Duncan’s multiple range test following one-way ANOVA, conducted with IBM SPSS 27 computational platform (SPSS Inc., Chicago, IL, USA) for hypothesis testing validation. Charts and tables were generated using OriginPro 2024b and WPS Office, respectively. The R package (R version 4.2.2) was utilized to generate a correlation thermograph.

## 3 Results and discussion

### 3.1 Influences of continuous cropping on the agronomic traits of *P. ternata*

**Figure 1:**
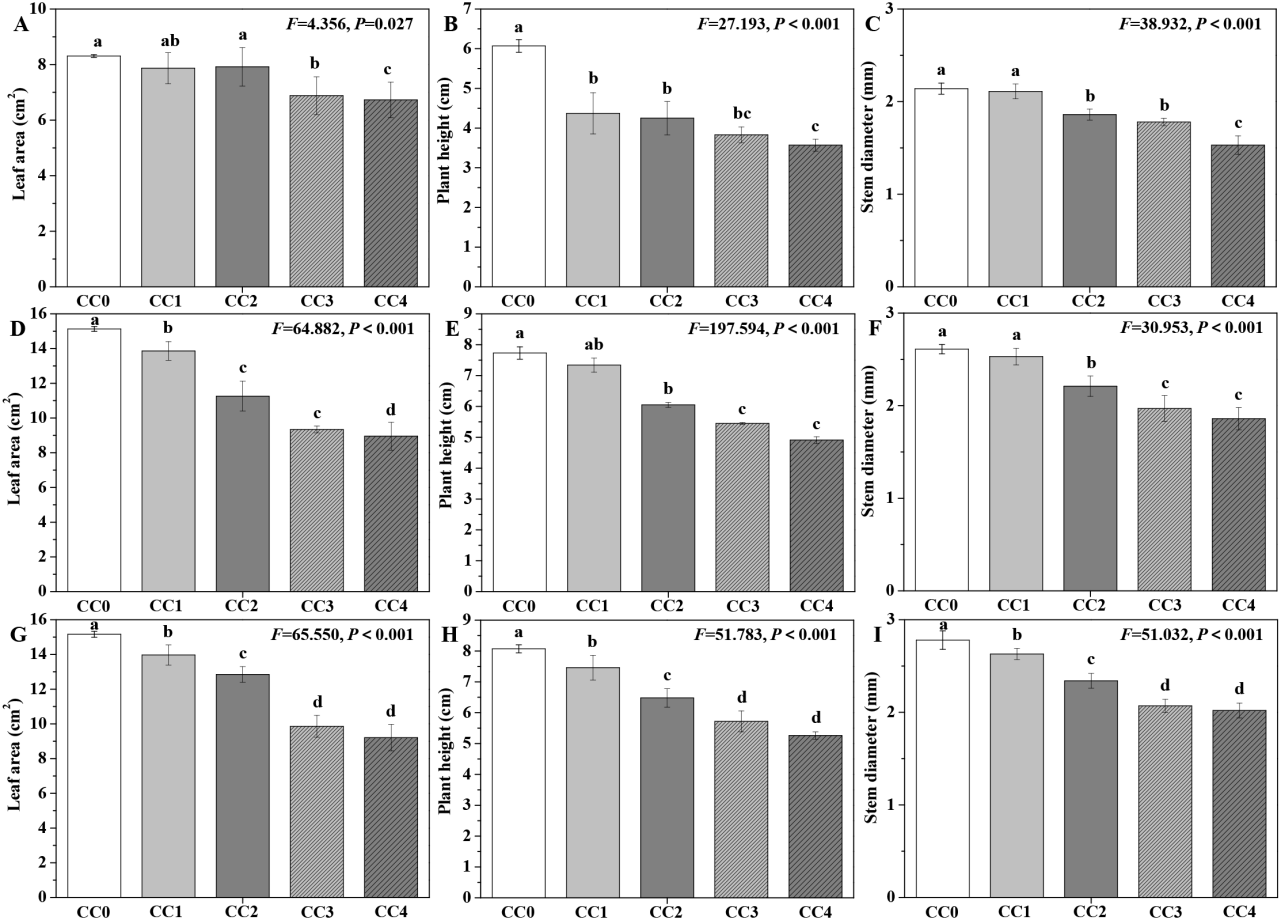
Influences of different continuous cropping years on the agronomic traits of *P. ternata*. CC0, CC1, CC2, CC3, and CC4 represent 0, 1, 2, 3, and 4 years of continuous cropping, respectively. **(A)** Leaf area (cm^2^) during full seedling stage; **(B)** plant height (cm) during full seedling stage; **(C)** stem diameter (cm) during full seedling stage; **(D)** leaf area (cm^2^) during vigorous growth stage; **(E)** plant height (cm) during vigorous growth stage; **(F)** stem diameter (cm) during vigorous growth stage; **(G)** leaf area (cm^2^) during sprout tumble stage; **(H)** plant height (cm) during inverted seedling stage; and **(I)** stem diameter (cm) during inverted seedling stage. Bar charts and error bars represent the mean and standard deviation of three biological replicates (*n* = 3). Different lowercase letters indicate statistically significant differences according to Duncan’s test (*p* < 0.05).

The influences of different continuous cropping years on the agronomic traits of *P. ternata* are shown in **Figure 1**. During three consecutive growing seasons, the leaf area, plant height, and stem diameter of *P. ternata* exhibited a progressive decline with increasing duration of continuous cropping. At the full seedling stage (**Figures 1A–C**), no significant differences in leaf area were found among the CC0, CC1, and CC2 treatments, though all demonstrated significantly (*p* < 0.05) greater values compared to the CC3 and CC4 treatments. Regarding plant height, CC1 and CC2 were statistically (*p* < 0.05) superior to CC4. While CC3 displayed no significant difference in plant height relative to CC1, CC2, and CC4, all four treatments (CC1–CC4) were significantly (*p* < 0.05) inferior to CC0. In terms of stem diameter, CC0 and CC1 displayed no significant differences and were significantly (*p* < 0.05) greater than those of CC2, CC3, and CC4. Additionally, CC2 and CC3 showed no significant difference but were statistically (*p* < 0.05) higher than CC4.

During the vigorous growth stage (**Figures 1D–F**), significant differences (*p* < 0.05) in leaf area of *P. ternata* were observed across all treatments (CC0–CC4). Additionally, CC0 and CC1 exhibited statistically (*p* < 0.05) comparable plant heights and stem diameters, both of which were significantly (*p* < 0.05) greater than those of CC2, CC3, and CC4. CC3 and CC4 showed no significant differences in plant height or stem diameter, but both parameters were significantly (*p* < 0.05) inferior to those of CC2. During the sprout tumble stage (**Figures 1G–I**), significant differences (*p* < 0.05) in agronomic traits were observed among CC0, CC1, CC2, and CC3 (or CC4) treatments, while no significant differences were found between CC3 and CC4. Chen et al. (2023) and Tian et al. (2024) indicated that continuous cropping for 2 years significantly reduced the leaf area, plant height, and stem diameter of *P. ternata*. Hang et al. (2023) reported that the leaf length and width of *P. ternata* showed significant differences between continuous cropping for 0 years and 1 or 2 years. These findings emphasize that continuous cropping for 1~4 years obviously inhibited the morphological development of *P. ternata*, extend the results from continuous cropping for 3~4 years, and further confirm that continuous cropping severely inhibited the growth of *P. ternata* plants.

### 3.2 Influences of continuous cropping on the emergence and sprout tumble of *P. ternata*

**Figure 2:**
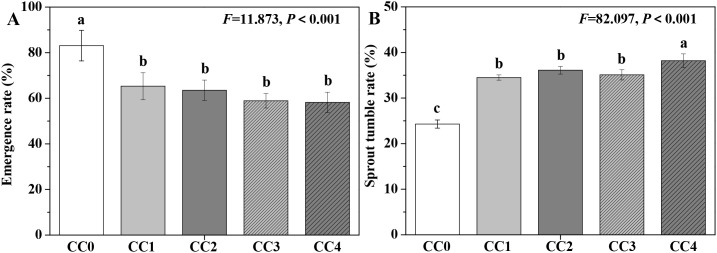
Influences of different continuous cropping years on the emergence rate **(A)** and sprout tumble rate **(B)** of *P. ternata*. CC0, CC1, CC2, CC3, and CC4 represent 0, 1, 2, 3, and 4 years of continuous cropping, respectively. Bar charts and error bars represent the mean and standard deviation of three biological replicates (*n* = 3), respectively. Different lowercase letters indicate statistically significant differences according to Duncan’s test (*p* < 0.05).

The influences of different continuous cropping years on the emergence rate and sprout tumble rate (a wilting phenomenon) of *P. ternata* are displayed in **Figure 2**. CC1, CC2, CC3, and CC4 significantly (*p* < 0.05) decreased the emergence rate of *P. ternata* and increased its inverted seedling rate compared to no continuous cropping (CC0). Compared with no continuous cropping (CC0), the emergence rates of *P. ternata* under 1~4 years of continuous cropping (CC1~CC4) decreased by 21.42%, 23.59%, 29.12%, and 29.96%, while the inverted seedling rates increased by 41.98%, 48.56%, 44.44%, and 57.20%, respectively. These results show that continuous cropping notably affected the emergence of *P. ternata* and inhibited its growth and development. Chen et al. (2023) and Tian et al. (2024) indicated that continuous cropping for 2 years increased the sprout tumble rate of *P. ternata*. Meanwhile, continuous cropping for 2 years also declined its emergence rate (Tian et al., 2024). Moreover, Hang et al. (2023) found that continuous cropping enhanced the earlier occurrence of the “sprout tumble” (i.e., inverted seedling phenomenon) in *P. ternata*. The results of this study further confirm that continuous cropping significantly affected the emergence of *P. ternata* and promoted its sprout tumble phenomenon.

### 3.3 Influences of continuous cropping on the photosynthetic capacity of *P. ternata*

**Figure 3:**
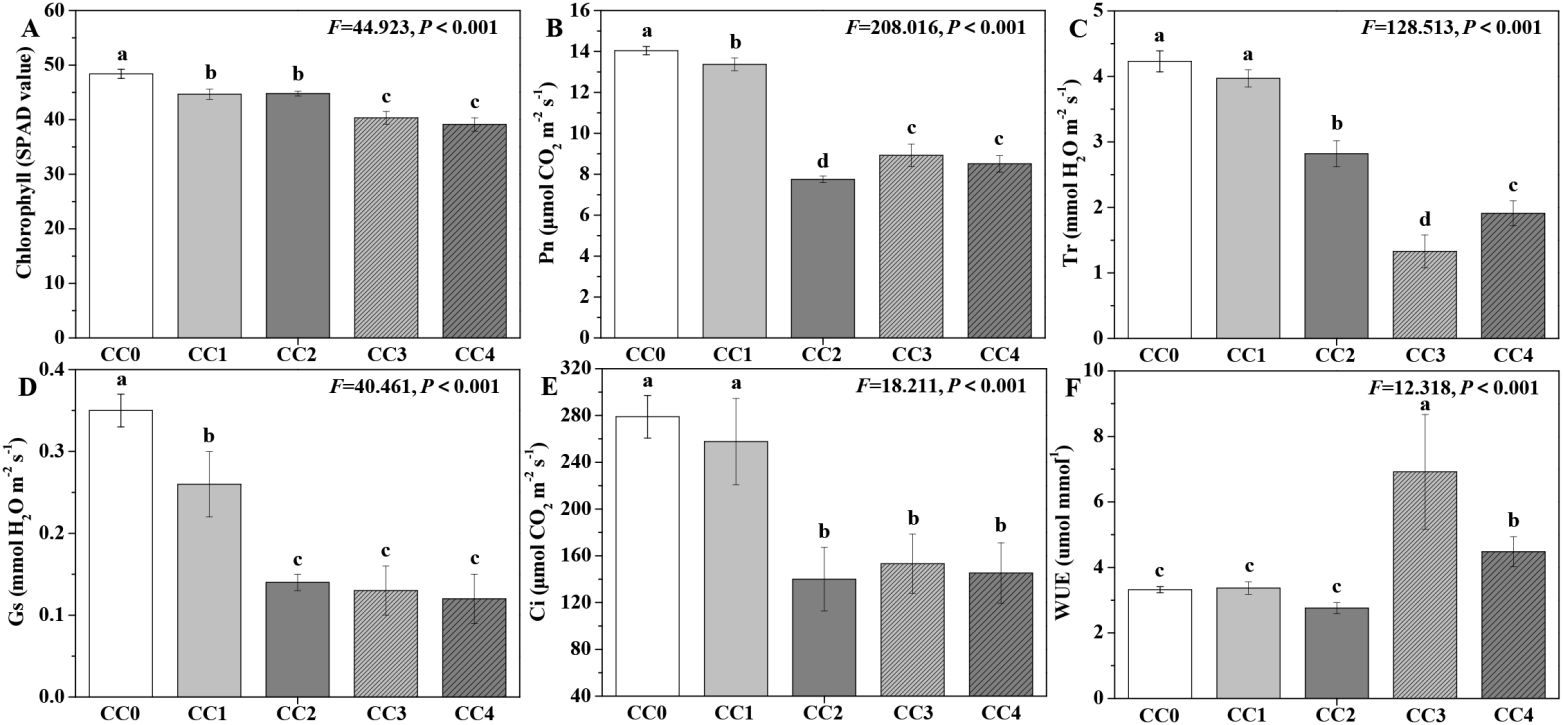
Influences of different continuous cropping years on the photosynthetic capacity of *P. ternata* during the vigorous growth stage. CC0, CC1, CC2, CC3, and CC4 represent 0, 1, 2, 3, and 4 years of continuous cropping, respectively. **(A)** Chlorophyll content (SPAD value), **(B)** photosynthetic rate (Pn; μmol CO_2_ m^−2^ s^−1^), **(C)** transpiration rate (Tr; mmol H_2_O m^−2^ s^−1^), **(D)** stomatal conductance (Gs; mmol H_2_O m^−2^ s^−1^), **(E)** intercellular carbon dioxide concentration (Ci; μmol CO_2_ m^−2^ s^−1^), and **(F)** water use efficiency (WUE; mmol mmol^−1^). Bar charts and error bars represent the mean and standard deviation of three biological replicates (*n* = 3). Different lowercase letters indicate statistically significant differences according to Duncan’s test (*p* < 0.05).

The influences of different continuous cropping years on the photosynthetic capacity of *P. ternata* in the vigorous growth stage are shown in **Figure 3**. The chlorophyll content, Pn, Tr, Gs, and Ci of *P. ternata* exhibited a declining tendency with increasing duration of continuous cropping (**Figures 3A–E**). Compared with no continuous cropping (CC0), CC1~CC4 significantly (*p* < 0.05) reduced the chlorophyll content, Pn, and Gs of *P. ternata*, while CC2~CC4 significantly (*p* < 0.05) decreased its Tr and Ci. The chlorophyll content, Pn, Tr, Gs, and Ci of *P. ternata* without continuous cropping (CC0) was 1.08, 1.08, 1.20, and 1.24 times; 1.05, 1.81, 1.57, and 1.65 times; 1.07, 1.50, 3.18, and 2.21 times; 1.35, 2.50, 2.69, and 2.92 times; and 1.08, 1.99, 1.82, and 1.92 times higher, respectively, compared to those in continuous cropping for 1~4 years (CC1~CC4). CC0, CC1, and CC2 showed no significant differences in the water use efficiency (WUE) of *P. ternata*, but all were significantly (*p* < 0.05) lower than that of CC3 or CC4 (**Figure 3F**), which might be because *P. ternata* could improve its water use efficiency to resist more severe continuous cropping adversity. The findings demonstrate that long-term continuous cropping significantly decreased the photosynthetic capacity of *P. ternata.* Hang et al. (2023) indicated that continuous cropping for 1 or 2 years reduced the chlorophyll *a*, chlorophyll *b*, and carotenoid contents of *P. ternata*, thus affecting its photosynthetic capacity. Chen et al. (2023) found that continuous cropping for 2 years decreased the Pn, Ci, Gs, and Tr of *P. ternata*. Tian et al. (2024) demonstrated that continuous cropping for 2 years also decreased their chlorophyll content and WUE. In general, continuous cropping gradually acidified the soil, and its nutrient or microbial community structure became increasingly sealed or unbalanced, which led to the poor growth of *P. ternata* plants, thereby resulting in low photosynthetic capacity. The results in this study also showed that continuous cropping notably inhibited the chlorophyll, Pn, Tr, Gs, and Ci of *P. ternata* and affected its photosynthetic production.

### 3.4 Influences of continuous cropping on the resistance of *P. ternata*

**Figure 4:**
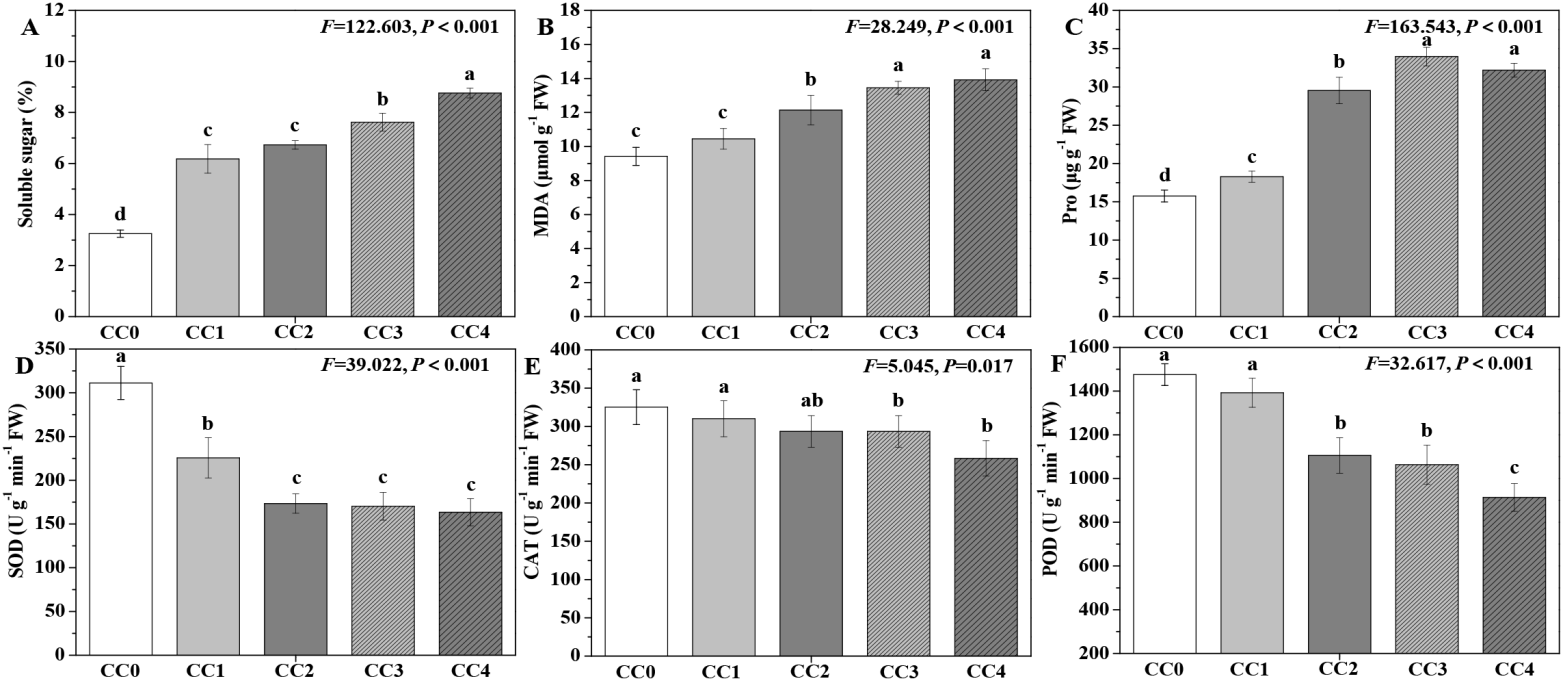
Influences of different continuous cropping years on the resistance-related substance contents and enzyme activities of *P. ternata* during the vigorous growth stage. CC0, CC1, CC2, CC3, and CC4 represent 0, 1, 2, 3, and 4 years of continuous cropping, respectively. **(A)** Soluble sugar (%), **(B)** malondialdehyde (MDA; μmol g^−1^ FW), **(C)** proline (Pro; μg g^−1^ FW), **(D)** superoxide dismutase (SOD; U g^−1^ min^−1^ FW), **(E)** catalase (CAT; U g^−1^ min^−1^ FW), and **(F)** peroxidase (POD; U g^−1^ min^−1^ FW). Bar charts and error bars represent the mean and standard deviation of three biological replicates (*n* = 3). Different lowercase letters indicate statistically significant differences according to Duncan’s test (*p* < 0.05).

The influences of different continuous cropping years on *P. ternata* resistance in the vigorous growth stage are displayed in **Figure 4**. The soluble sugar, MDA, and proline (Pro) contents exhibited an increasing trend with the duration of continuous cropping (**Figures 4A–C**), while its SOD, CAT, and POD activities displayed a declining trend (**Figures 4D–F**). Compared with no continuous cropping (CC0), CC1~CC4 significantly (*p* < 0.05) increased the soluble sugar and Pro contents, and CC2~CC4 significantly (*p* < 0.05) increased the MDA content. The soluble sugar, MDA, and Pro contents of *P. ternata* under continuous cropping for 1~4 years (CC1~CC4) increased by 47.41%~62.90%, 11.58%~33.62%, and 13.84%~51.07%, respectively, compared to CC0. Compared with no continuous cropping (CC0), CC1~CC4 significantly (*p* < 0.05) decreased the SOD activity of *P. ternata*, while CC3 and CC4 (*p* < 0.05) reduced its CAT activity, and CC2~CC4 significantly (*p* < 0.05) reduced its POD activity. The SOD, CAT, and POD activities of *P. ternata* with continuous cropping for 1–4 years (CC1~CC4) decreased by 27.51%~47.49%, 3.17%~36.47%, and 4.63%~20.56%, respectively, compared to CC0. Chen et al. (2023) and Tian et al. (2024) reported that 2 years of continuous cropping increased soluble sugar, MDA, and Pro contents while reducing SOD, CAT, and POD activities. Hang et al. (2023) also demonstrated that continuous cropping significantly reduced POD and SOD activities. Generally, although plant growth is poor and reactive oxygen species (ROS) metabolism is heightened under strong continuous cropping stress, the activities of SOD, CAT, and POD would be expected to increase during this time. However, it should be recognized that SOD activity levels are an intrinsic characteristic of the plant itself, and some plants may naturally exhibit high SOD activity while maintaining good growth. Meanwhile, adversity stress may only stimulate an increase in SOD activity to a certain extent. The findings of this study indicate that long-term continuous cropping significantly increased the contents of resistance-related substances in *P. ternata* while reducing its antioxidant enzyme activities. These promoting or inhibiting effects became more pronounced with the extension of continuous cropping years.

### 3.5 Influences of continuous cropping on the electrophysiological information of *P. ternata*

The influences of different continuous cropping years on the *electrical signals* of *P. ternata* in the vigorous growth stage are shown in **Table 1**. The IR, IZ, IXL, and IXc of *P. ternata* exhibited a gradual increasing tendency with the increasing duration of continuous cropping, while its IC showed an increasingly declining tendency. Compared with no continuous cropping (CC0), CC2~CC4 significantly (*p* < 0.05) increased the IR of *P. ternata*, CC3 and CC4 (*p* < 0.05) increased its IZ, and CC4 significantly (*p* < 0.05) raised its IXL and IXc. In contrast, CC1~CC4 significantly (*p* < 0.05) decreased its IC. The IR, IZ, IXL, and IXc of *P. ternata* subjected to 1~4 years of continuous cropping (CC1~CC4) increased by 31.73%~70.24%, 17.98%~50.41%, 26.82%~58.12%, and 30.17%~68.99%, respectively, compared to CC0, while their IC decreased by 26.84%~57.97%. In general, well-grown plants exhibit high capacitance and low resistance characteristics (Nguyen et al., 2018; Szechyńska-Hebda et al., 2017). Tian et al. (2024) found that continuous cropping for 2 years notably increased the IR, IZ, IXL, and IXc of *P. ternata* and decreased its IC. The results indicate that long-term continuous cropping increased the IR, IZ, IXL, and IXc of *P. ternata* and decreased its IC, with these promoting or inhibiting effects becoming more pronounced with the extension of continuous cropping years. This further confirms that continuous cropping significantly affects its growth and development.

**Table 1:** The influences of different continuous cropping years on IR, IZ, IXL, IXc, and IC of *P. ternata* during the vigorous growth stage.

CC years	IR (MΩ)	IZ (MΩ)	IXL (MΩ)	IXc (MΩ)	IC (pF)
CC0	1.52 ± 0.43 d	0.29 ± 0.02 d	0.25 ± 0.01 b	1.68 ± 0.44 b	211.77 ± 7.66 a
CC1	2.22 ± 0.26 cd	0.35 ± 0.01 c	0.34 ± 0.01 b	2.40 ± 0.27 b	154.92 ± 3.65 b
CC2	2.94 ± 0.84 bc	0.38 ± 0.02 c	0.39 ± 0.02 b	3.17 ± 0.82 ab	135.82 ± 5.37 b
CC3	3.99 ± 1.07 ab	0.46 ± 0.04 b	0.47 ± 0.05 ab	4.00 ± 1.14 ab	114.76 ± 13.75 bc
CC4	5.09 ± 0.17 a	0.58 ± 0.01 a	0.57 ± 0.01 a	5.40 ± 0.20 a	89.01 ± 0.99 c
*F*-values	14.133	0.702	8.854	13.783	111.111
*p-*values	< 0.001	< 0.001	< 0.001	< 0.001	< 0.001

CC0, CC1, CC2, CC3, and CC4 represent 0, 1, 2, 3, and 4 years of continuous cropping, respectively. Data display the mean ± standard deviation of three biological replicates (*n* = 3); different lowercase letters denote the statistically significant differences according to Duncan’s test (*p* < 0.05). *IR*, intrinsic resistance; *IZ*, intrinsic impedance; *IXL*, intrinsic inductive reactance; *IXc*, intrinsic capacitive reactance; *IC*, intrinsic capacitance.

**Figure 5:**
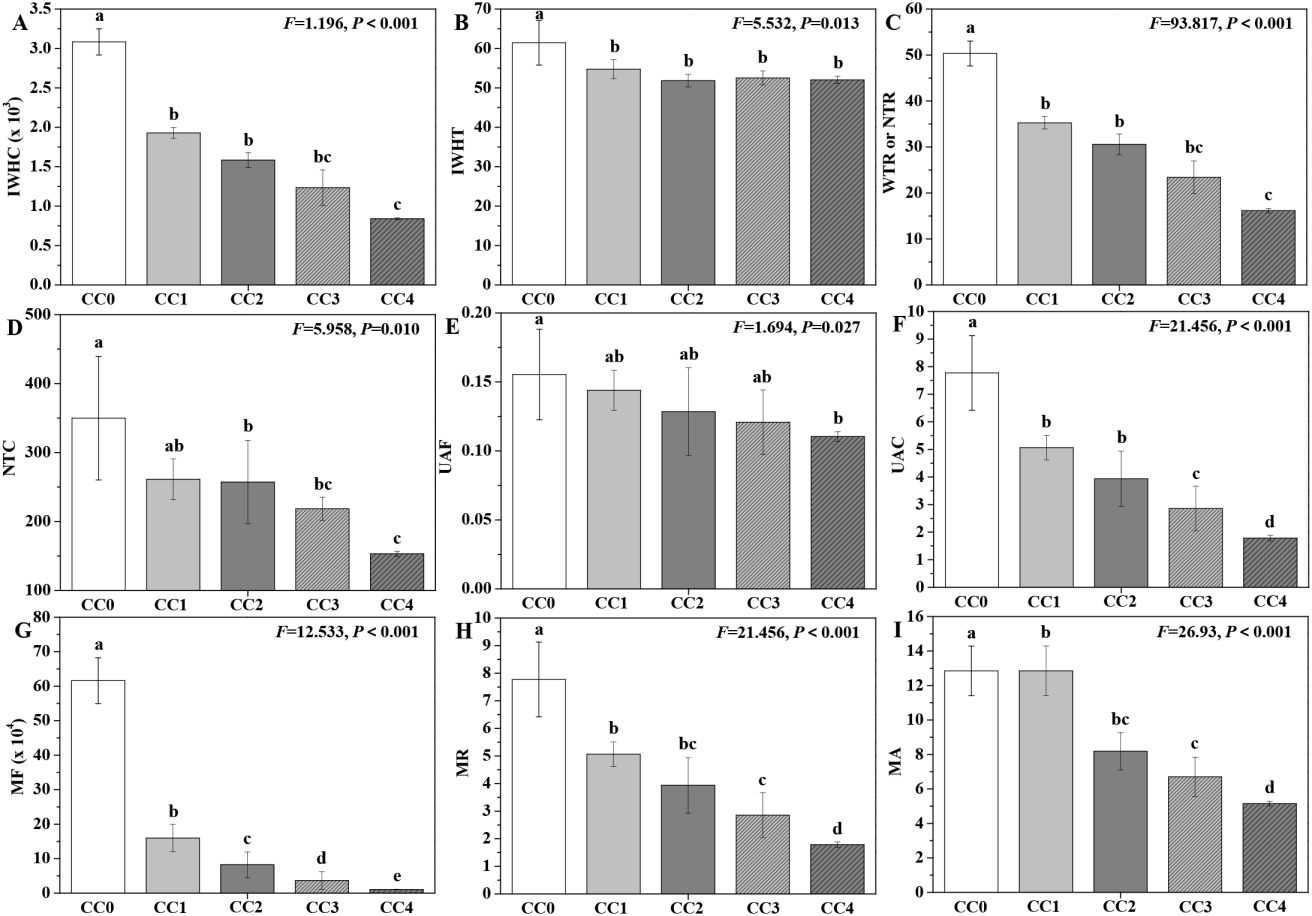
Influences of different continuous cropping years on intracellular water metabolism, nutrient transport, and metabolic activity in *P. ternata* during the vigorous growth stage. CC0, CC1, CC2, CC3, and CC4 represent 0, 1, 2, 3, and 4 years of continuous cropping, respectively. **(A)** Intracellular water-holding capacity (IWHC), **(B)** intracellular water-holding time (IWHT), **(C)** water (WTR) or nutrient transfer rate (NTR), **(D)** nutrient transport capacity (NTC), **(E)** active transport flow of nutrient (UAF), **(F)** nutrient active transport capacity (NAC), **(G)** metabolic flow (MF), **(H)** metabolic rate (MR), and **(I)** metabolic activity (MA). Bar charts and error bars represent the mean and standard deviation of three biological replicates (*n* = 3). Different lowercase letters indicate statistically significant differences according to Duncan’s test (*p* < 0.05).

The influences of different continuous cropping years on the intracellular water metabolism, nutrient transport, and metabolic activity of *P. ternata* in the vigorous growth stage are displayed in **Figure 5**. The IWHC, IWHT, WTR or NTR, NTC, UAF, NAC, MF, MR, and MA of *P. ternata* exhibited a gradual declining trend with increasing duration of continuous cropping. Compared with no continuous cropping (CC0), CC1~CC4 significantly (*p* < 0.05) decreased the IWHC, IWHT, WTR or NTR, NAC, MF, MR, and MA of *P. ternata*; CC2~CC4 (*p* < 0.05) decreased its NTC; and CC4 significantly (*p* < 0.05) decreased its UAF. CC1~CC4 showed no significant differences in IWHT or UAF; CC1~CC3 exhibited no significant differences in IWHC, WTR or NTR, or NTC; CC1 and CC2 displayed no significant differences in UAC, MR, or MA; and CC2 and CC3 showed no significant differences in MR or MA. Moreover, the IWHC, WTR or NTR, and NTC of *P. ternata* with continuous cropping for 1–2 years (CC1~CC2) were significantly (*p* < 0.05) higher than those of CC4, and their UAC was also significantly (*p* < 0.05) higher than that of CC3 or CC4. The MR and MA of *P. ternata* with 1 year of continuous cropping (CC1) were significantly (*p* < 0.05) higher than those in CC3 or CC4. Meanwhile, there was a significant difference (*p* < 0.05) in MF of *P. ternata* among all treatments.

Previous reports used intracellular water metabolism, nutrient transport, and plant metabolic activity to accurately and reliably evaluate the physiological status of *Pseudostellaria heterophylla*, *Orychophragmus violaceus*, *Brassica napus*, etc (Tian et al., 2025; Zhang et al., 2020, 2021a, 2021b). Tian et al. (2024) also found that continuous cropping for 2 years notably decreased the intracellular water metabolism, nutrient transport, and metabolic activity of *P. ternata*. The results in this study demonstrate that short-term continuous cropping dramatically inhibited the intracellular water metabolism, nutrient transport, and metabolic activity of *P. ternata*, and that this inhibitory effect intensified with longer durations of continuous cropping, further supporting the conclusion that continuous cropping significantly restricts its growth and development.

### 3.6 Influences of continuous cropping on the medicinal composition and tuber productivity of *P. ternata*

The influences of different continuous cropping years on the medicinal composition and tuber productivity of *P. ternata* are shown in **Table 2**. With increasing duration of continuous cropping, the medicinal composition and tuber productivity parameters first declined and then increased. Compared with no continuous cropping (CC0), CC1~CC4 significantly (*p* < 0.05) reduced the ash, butanedioic acid, extractum, total yield, medicinal material yield, and seed yield of *P. ternata*. Additionally, CC2 significantly (*p* < 0.05) reduced its water content. The water, ash, extractum, butanedioic acid, medicinal material yield, seed yield, and total yield of *P. ternata* with continuous cropping for 1~4 years (CC1~CC4) decreased by 0.99%~15.34%, 8.31%~16.89%, 9.35%~13.03%, 31.10%~36.12%, 25.26%~59.89%, 18.91%~56.75%, and 23.84%~59.20%, respectively, compared to CC0. Moreover, among CC1~CC4, the quality and yield parameters of *P. ternata* followed an approximate order: CC2 < CC3 < CC4 < CC1. Chen et al. (2023) and Tian et al. (2024) reported that continuous cropping for 2 years reduced the quality and yield of *P. ternata* tubers. In this study, the findings show that continuous cropping notably reduced the quality and yield of *P. ternata*. Meanwhile, continuous cropping for 3~4 years may promote its quality and yield compared to 1~2 years of continuous cropping, possibly due to the enhanced adaptability of *P. ternata* to prolonged continuous cropping stress.

**Table 2:** The influences of different continuous cropping years on the quality and yield of *P. ternata* tubers.

CC years	Water (%)	Ash (%)	Extractum (%)	Butanedioic acid (%)	Medicinal material yield (kg hm^−2^)	Seed yield (kg hm^−2^)	Total yield (kg hm^−2^)
CC0	10.13 ± 0.22 a	3.73 ± 0.07 a	19.57 ± 0.62 a	0.39 ± 0.01 a	2,864.04 ± 14.91 a	806.28 ± 14.64 a	3,670.32 ± 27.31 a
CC1	10.03 ± 0.35 a	3.42 ± 0.11 b	17.74 ± 0.53 b	0.27 ± 0.00 b	2,141.56 ± 56.19 b	653.83 ± 23.55 b	2,795.39 ± 33.08 b
CC2	8.57 ± 0.27 b	3.10 ± 0.08 c	17.06 ± 0.61 b	0.25 ± 0.01 c	1,148.71 ± 41.43 e	348.71 ± 19.24 c	1,497.42 ± 59.42 e
CC3	9.92 ± 0.17 a	3.31 ± 0.12 b	17.02 ± 0.96 b	0.25 ± 0.01 c	1,376.08 ± 34.49 d	359.55 ± 14.56 c	1,735.63 ± 43.06 d
CC4	10.01 ± 0.27 a	3.27 ± 0.05 b	17.41 ± 0.74 b	0.25 ± 0.01 c	1,838.51 ± 45.30 c	627.31 ± 25.18 b	2,465.82 ± 32.75 c
*F*-values	18.529	21.621	6.634	333.344	821.784	299.997	1,367.197
*p*-values	< 0.001	< 0.001	0.007	< 0.001	< 0.001	< 0.001	< 0.001

CC0, CC1, CC2, CC3, and CC4 represent 0, 1, 2, 3, and 4 years of continuous cropping, respectively. Data display the mean ± standard deviation of three biological replicates (*n* = 3). Different lowercase letters indicate statistically significant differences according to Duncan’s test (*p* < 0.05).

### 3.7 Influences of continuous cropping on the growth and obstacle indices of *P. ternata*

**Figure 6:**
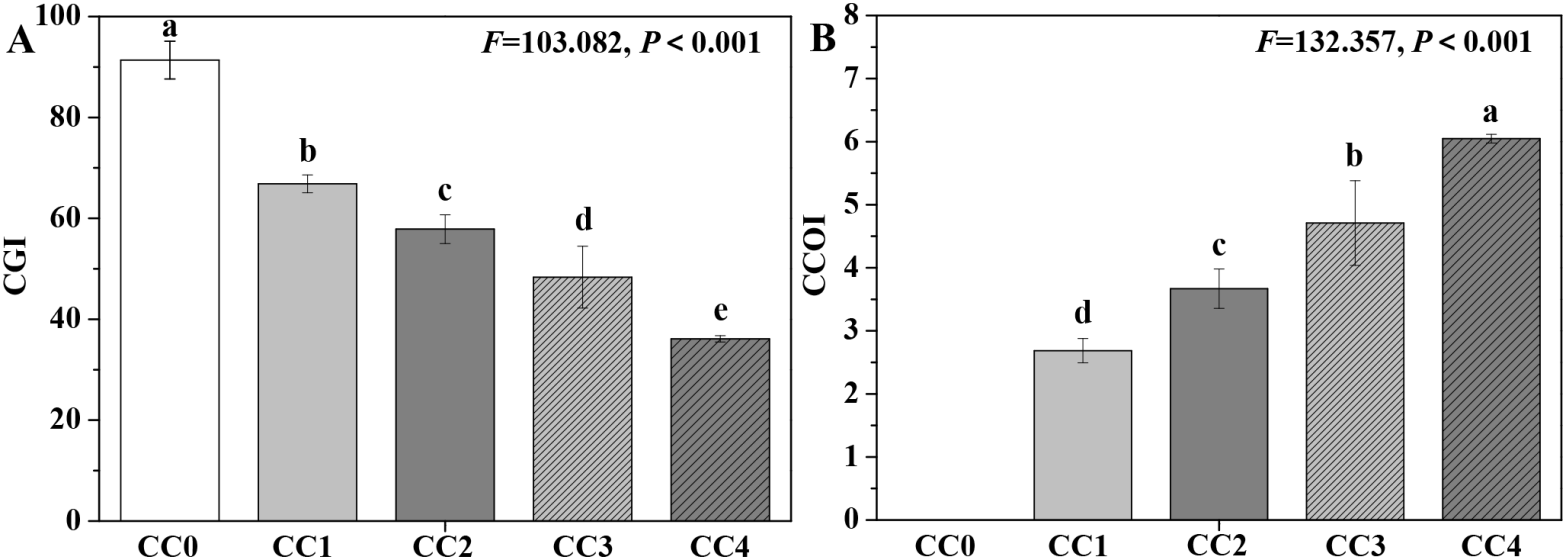
Influences of different continuous cropping years on the growth and obstacle indices of *P. ternata* during the vigorous growth stage. CC0, CC1, CC2, CC3, and CC4 represent 0, 1, 2, 3, and 4 years of continuous cropping, respectively. **(A)** Comprehensive growth index (CGI) and **(B)** continuous cropping obstacle index (CCOI). Bar charts and error bars represent the mean and standard deviation of three biological replicates (*n* = 3). Different lowercase letters indicate statistically significant differences according to Duncan’s test (*p* < 0.05).

The influences of different continuous cropping years on the growth and obstacle indices of *P. ternata* in the vigorous growth stage are shown in **Figure 6**. The CGI of *P. ternata* showed a progressively decreasing trend with increasing duration of continuous cropping, while its CCOI exhibited a gradually increasing trend. The CGI values for continuous cropping years 1~4 (CC1~CC4) were 66.84, 57.86, 48.35, and 36.11, respectively, all significantly (*p* < 0.05) lower than that of no continuous cropping (CC0 = 91.38). Meanwhile, there were significant differences (*p* < 0.05) in CGI among CC1, CC2, CC3, and CC4. Moreover, the CCOI of *P. ternata* under continuous cropping for 1–4 years (CC1~CC4) were 2.69, 3.67, 4.71, and 6.05, respectively, with significant differences observed among them (*p* < 0.05). These findings emphasize that as the duration of continuous cropping increased, the growth inhibition effect on *P. ternata* gradually intensified, and the continuous cropping obstacle stress became progressively more severe. The CGI and CCOI indices, based on the plant’s electrical information, effectively characterized the growth and obstacle stress status of continuously cropped *P. ternata*.

### 3.8 Correlation analysis

**Figure 7:**
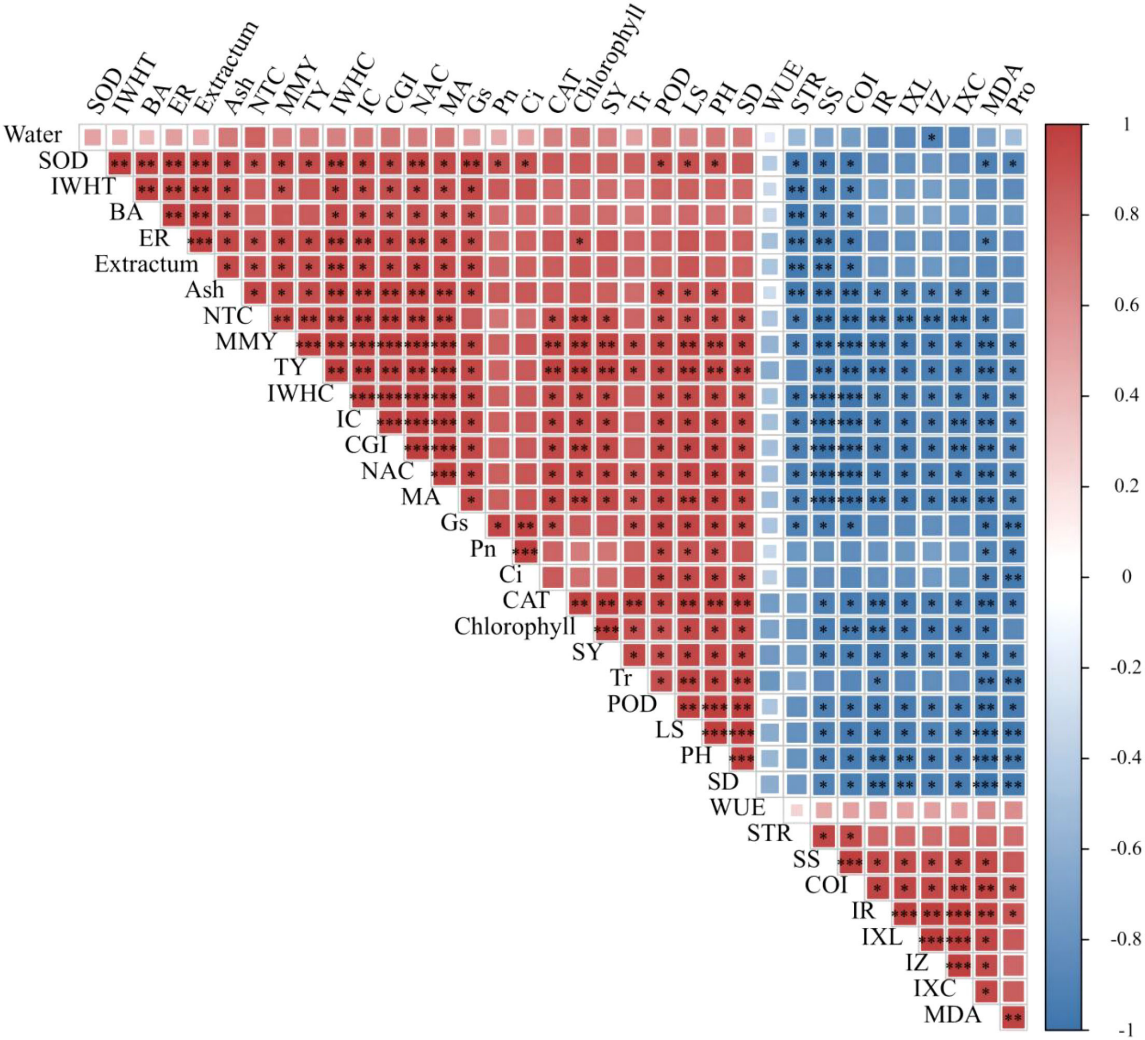
Correlation analysis between growth or obstacle indices and agronomic traits, photosynthesis, resistance, electrophysiology, quality, and yield parameters. PH, plant height; LA, leaf area; SD, stem diameter; BA, butanedioic acid; ER, emergence rate; STR, sprout tumble rate; SS, soluble sugar; MMY, medicinal material yield; SY, seed yield; TY, total yield. ^***^ *p* < 0.001, ^**^ *p* < 0.01, and ^*^ *p* < 0.05 indicate significant correlations at 0.001, 0.01, and 0.05 levels, respectively.

Pearson’s correlation analysis was applied to establish a correlation matrix between growth or obstacle indices and agronomic traits, photosynthesis, resistance, electrophysiology, quality, and yield parameters. As shown in **Figure 7**, the CGI of *P. ternata* exhibited significant (*p* < 0.05) synergistic correlations with its SOD, IWHT, butanedioic acid, emergence rate, extractum, Gs, CAT, seed yield, POD, leaf area, plant high, and stem diameter; significant (*p* < 0.01) synergistic correlations with its ash, NTC, total yield, and chlorophyll; and significant (*p* < 0.001) synergistic correlations with its IWHC, IC, NAC, and MA. Moreover, CGI exhibited significant (*p* < 0.05) antagonistic correlations with sprout tumble rate, IR, IXL, IZ, and proline; significant (*p* < 0.01) antagonistic correlations with IXc and MDA; and significant (*p* < 0.001) antagonistic correlations with soluble sugar and CCOI. Meanwhile, the CCOI of *P. ternata* exhibited significant (*p* < 0.05) antagonistic correlations with its SOD, IWHT, butanedioic acid, extractum, Gs, CAT, seed yield, POD, leaf area, plant high, and stem diameter; significant (*p* < 0.01) antagonistic correlations with its ash, NTC, total yield, and chlorophyll; and significant (*p* < 0.001) antagonistic correlations with its IWHC, IC, CGI, NAC, and MA. Additionally, CCOI exhibited significant (*p* < 0.05) synergistic correlations with sprout tumble rate, IR, IXL, IZ, and proline; significant (*p* < 0.01) synergistic correlations with IXc and MDA; and significant (*p* < 0.001) synergistic correlations with soluble sugar. These findings highlight that the CGI and CCOI indices, based on the plant’s electrophysiological information, showed strong correlations with agronomic traits, photosynthesis, resistance, electrophysiological information, quality, and yield, demonstrating their utility in accurately characterizing the growth and obstacle stress status of continuous cropping *P. ternata*.

The plant’s electrical information is considered the fastest physiological response to abiotic or biotic stimuli (Favre et al., 2011; Gallé et al., 2015; Sukhov et al., 2015; Xing et al., 2021; Zhang et al., 2015). Electrophysiological parameters based on plant electrical information have been applied to accurately and reliably evaluate the physiological status of various plants, such as *Pseudostellaria heterophylla*, *Camellia sinensis*, *Lonicera japonica*, *Parthenocissus quinquefolia*, *Solanum lycopersicum*, *Broussonetia papyrifera*, and *Morus alba*, among others (Tian et al., 2025; Wei et al., 2024; Xie et al., 2021; Xing et al., 2021, 2022; Zhang et al., 2020, 2021a, 2021b; Zhao et al., 2023). In this study, the CGI of *P. ternata* displayed a progressively declining trend with increasing duration of continuous cropping, while its CCOI exhibited a gradually increasing trend. The CGI under continuous cropping for 1~4 years was significantly (*p* < 0.05) lower than that of no continuous cropping, and the CCOI showed significant (*p* < 0.05) differences among the 1~4 years of continuous cropping. Meanwhile, CGI and CCOI, based on the plant’s electrophysiological information, exhibited good correlations with the agronomic traits, photosynthesis, resistance, quality, and yield parameters of *P. ternata*. These findings support that CGI and CCOI can accurately, rapidly, and reliably characterize the growth and obstacle stress status of *P. ternata* under continuous cropping. Overall, this study systematically reveals the physiological and electrophysiological responses of *P. ternata* to long-term continuous cropping and provides a novel approach based on the plant’s electrical information for rapid monitoring of continuous cropping obstacle occurrence.

## 4 Conclusions

In summary, continuous cropping notably inhibited the morphological development, photosynthesis, resistance, quality, and yield of *P. ternata.* Meanwhile, it markedly suppressed IC, intracellular water metabolism, nutrient transport, and metabolic activity, while increasing IR, IZ, IXL, and IXc. Moreover, these inhibitory or promotive effects intensified with the increasing duration of continuous cropping. Notably, CGI and CCOI, based on the plant’s electrical information, showed strong correlations with the agronomic traits, photosynthesis, resistance, quality, and yield parameters of *P. ternata*, accurately and rapidly characterizing its growth and continuous cropping obstacle status. This work systematically describes the physiological and electrophysiological responses of *P. ternata* to long-term continuous cropping and develops a novel approach using plant electrical information for rapid monitoring of continuous cropping obstacle occurrence.

## Data Availability

The original contributions presented in the study are included in the article/**Supplementary Material**. Further inquiries can be directed to the corresponding author.
